# Neotropical mammal diversity and the Great American Biotic Interchange: spatial and temporal variation in South America's fossil record

**DOI:** 10.3389/fgene.2014.00451

**Published:** 2015-01-05

**Authors:** Juan D. Carrillo, Analía Forasiepi, Carlos Jaramillo, Marcelo R. Sánchez-Villagra

**Affiliations:** ^1^Paläontologisches Institut und Museum, University of ZurichZurich, Switzerland; ^2^Smithsonian Tropical Research InstitutePanama City, Panama; ^3^Instituto Argentino de Nivología, Glaciología y Ciencias Ambientales (IANIGLA), CCT-CONICET MendozaMendoza, Argentina

**Keywords:** Miocene, Pliocene, biogeography, mammalia, South America

## Abstract

The vast mammal diversity of the Neotropics is the result of a long evolutionary history. During most of the Cenozoic, South America was an island continent with an endemic mammalian fauna. This isolation ceased during the late Neogene after the formation of the Isthmus of Panama, resulting in an event known as the Great American Biotic Interchange (GABI). In this study, we investigate biogeographic patterns in South America, just before or when the first immigrants are recorded and we review the temporal and geographical distribution of fossil mammals during the GABI. We performed a dissimilarity analysis which grouped the faunal assemblages according to their age and their geographic distribution. Our data support the differentiation between tropical and temperate assemblages in South America during the middle and late Miocene. The GABI begins during the late Miocene (~10–7 Ma) and the putative oldest migrations are recorded in the temperate region, where the number of GABI participants rapidly increases after ~5 Ma and this trend continues during the Pleistocene. A sampling bias toward higher latitudes and younger records challenges the study of the temporal and geographic patterns of the GABI.

## Introduction

The Neotropics [Neotropical region *sensu lato* of Morrone (2014)] supports an extremely large diversity of living mammals. Currently there are around 1500 recognized species which represent in the order of 30% of the total world mammal diversity. Included are endemic groups such as marsupials (opossums), xenarthrans (sloths, armadillos, and anteaters), caviomorph rodents (capybaras, spiny rats, chinchillas), platyrrhine monkeys, and phyllostomid bats (Patterson and Costa, 2012). The variety of biomes found in the Neotropics (lowland rainforest, savannas, mountain forest, scrublands, and deserts) could provide a partitioned environment enhancing species richness (Tews et al., 2004).

The current Neotropical mammal fauna is the result of a long evolutionary history. The Cenozoic (66–0 Ma) in South America was characterized by long term geographical isolation with the evolution of an endemic fauna (Simpson, 1980). Sporadic dispersal events from other geographic areas interrupted this isolation introducing novel clades into South America including caviomorph rodents during the middle Eocene (~41 Ma) and platyrrhine monkeys during the late Oligocene (~26 Ma) (Pascual, 2006; Antoine et al., 2012; Croft, 2012; Goin et al., 2012). The isolation of South America's mammal fauna ceased by ~10–7 Ma, when proximity, and then permanent connection was established with Central America. This connection initiated a massive faunal exchange between North America (NA) and South America (SA). This event is known as the Great American Biotic Interchange (GABI) (Simpson, 1980; Webb, 1985). The classic interpretation places the onset of the GABI by ~3.0 Ma, with some early migrations during the late Miocene from SA to NA by ~9 Ma and from NA to SA by ~7 Ma. Other studies using dated molecular phylogenies across a wide range of taxa indicate an important part of the interchange may have predated the permanent land connection by ~3 Ma (Koepfli et al., 2007; Cody et al., 2010; Eizirik et al., 2010; Eizirik, 2012). The core of the GABI is composed by a series of major migration “waves” during the Pliocene–Pleistocene (2.5–0.012 Ma) (Webb, 2006; Woodburne, 2010). Recently, several NA mammals have been reported from the late Miocene deposits, ~10 Ma, within the Amazon basin. These include a dromomerycine artiodactyl, gomphotheres, peccaries, and tapirs which suggest a more intense earlier connection (Campbell et al., 2000, 2010; Frailey and Campbell, 2012; Prothero et al., 2014). However, the taxonomy and age of some of these fossils have been questioned (Alberdi et al., 2004; Lucas and Alvarado, 2010; Lucas, 2013). In Amazonia, Pleistocene terraces are built from older Cenozoic deposits (Latrubesse et al., 1997), resulting in non-contemporaneous associations (Cozzuol, 2006). Even with these concerns in mind, in the last decades the presence of northern forms in South America is becoming better understood.

During the late Miocene (11.6–5.3 Ma) and early Pliocene (5.3–3.6 Ma), the GABI was taxonomically balanced, as predicted by the MacArthur–Wilson species equilibrium hypothesis, with similar number of NA and SA families participating in the interchange (Webb, 1976; Marshall et al., 1982). During the Pleistocene, NA mammals appeared to have diversified exponentially in SA, resulting in an overall prevalence of NA over SA–derived mammals. This could be the result of competitive displacement (Webb, 1976, 1991; Marshall et al., 1982), but this has not been subjected to rigorous analyses. In contrast, ecological replacement has been demonstrated for extinct metatherians and placental carnivores (Prevosti et al., 2013).

Vrba (1992) analyzed the GABI in the context of the “habitat theory” (i.e., physical environmental changes are the main drivers of “distribution drift”) and highlighted the importance of environmental changes over biotic interactions as the major cause of the biotic turnover. Webb (1991) proposed that the Pleistocene glaciations and the widespread development of savannas in the Neotropics facilitated dispersals during the GABI of savanna-adapted mammals. Woodburne (2010) agreed with Webb's model and related the pulses of faunistic movements to the glaciations and sea level changes of the Pliocene and Pleistocene. However, most recent evidence does not support the widespread expansion of savannas in the tropics during glacial times (Behling et al., 2010). The GABI was dynamic with bidirectional migrations (Carlini et al., 2008b; Castro et al., 2014) and with reciprocal exchanges within a single lineage (e.g., procyonids; Baskin, 1989; Forasiepi et al., 2014; and felids; Prevosti, 2006).

Potential biogeographic barriers or corridors along with environmental changes controlled patterns of movements (Webb, 1991; Woodburne, 2010). The Andes are currently an important biogeographic feature in South America extending for about 8000 km from Venezuela to Argentina, reaching average heights of about 4000 masl and maximum elevations up to 7000 masl (Ramos, 1999). The present day elevations of the northern and the north central Andes (north of 20°S) were reached during or soon after the late Miocene (Mora et al., 2009) and may have constituted a colonization corridor during the GABI (Patterson et al., 2012 and references therein).

**Figure 1:**
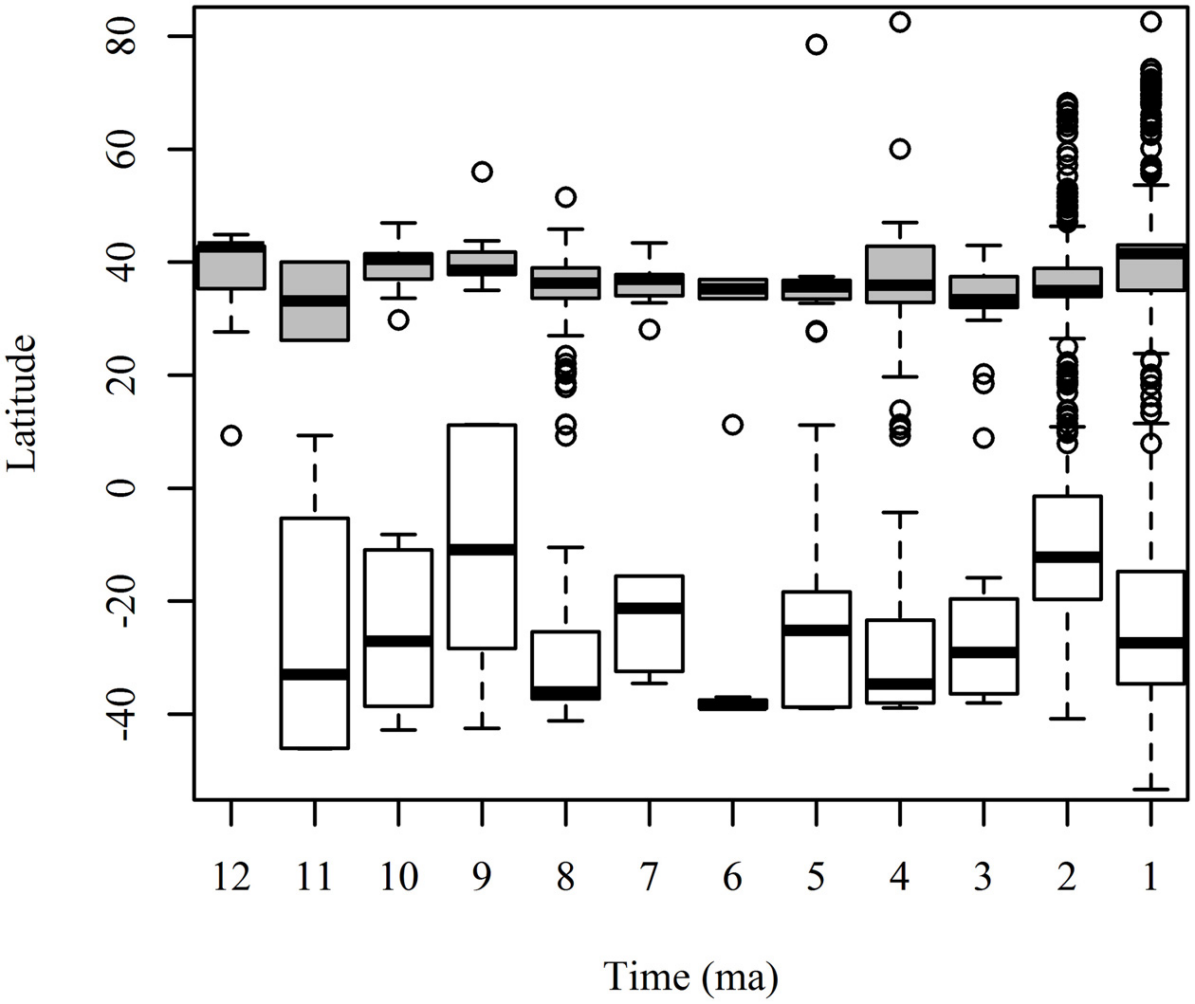
**Number of collections in the Paleobiology Database (PBDB) across latitude for land mammals in North America (gray boxes) and South America (white boxes) for each 1 ma period in the last 12 ma**. The boxplot shows the mean and standard deviation of the latitude of the PBDB collections for each time interval.

A full understanding of the GABI is difficult because of the difference in fossil sampling between low and high latitudes (Figure 1). Even with the major recent advances in Neotropical paleontology (Kay et al., 1997; Campbell, 2004; MacFadden, 2006; Sánchez-Villagra et al., 2010; Antoine et al., 2012), our knowledge of this large portion of territory that comprises the neotropics, twice the size of Europe and almost as large as North America is scarce (Croft, 2012).

In this contribution, we investigate biogeographic patterns for the middle and late Miocene (15.9–5.3 Ma) in SA at the initiation of the GABI. We review the temporal and geographical distribution of fossil mammals during the GABI and discuss the special significance of the fossil record from northern SA to understand the patterns and dynamics of the interchange.

## Materials and methods

**Figure 2:**
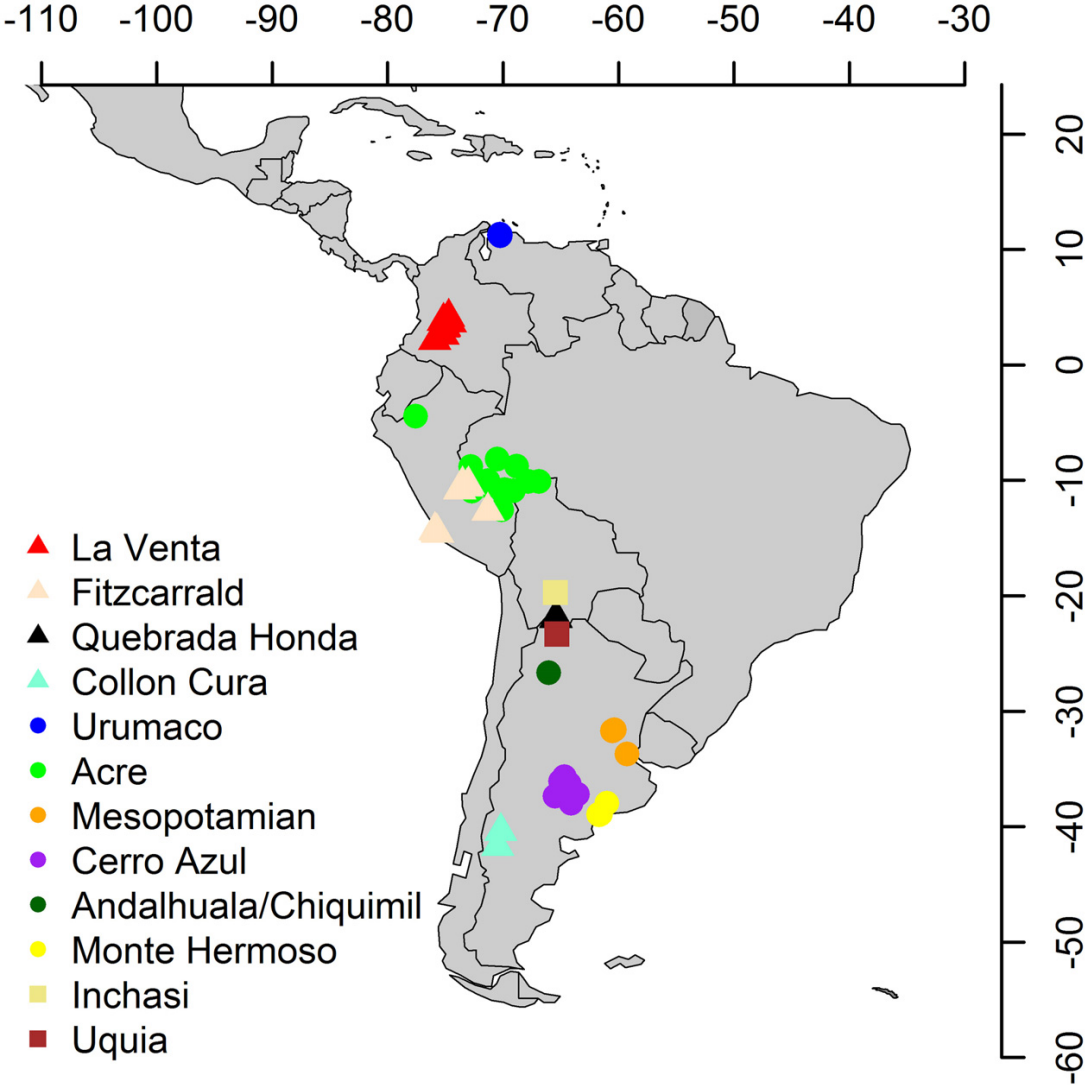
**Middle and late Miocene – Pliocene main fossil sites for land mammals in South America**. Triangles, middle Miocene; circles, late Miocene; squares, Pliocene.

**Figure 3:**
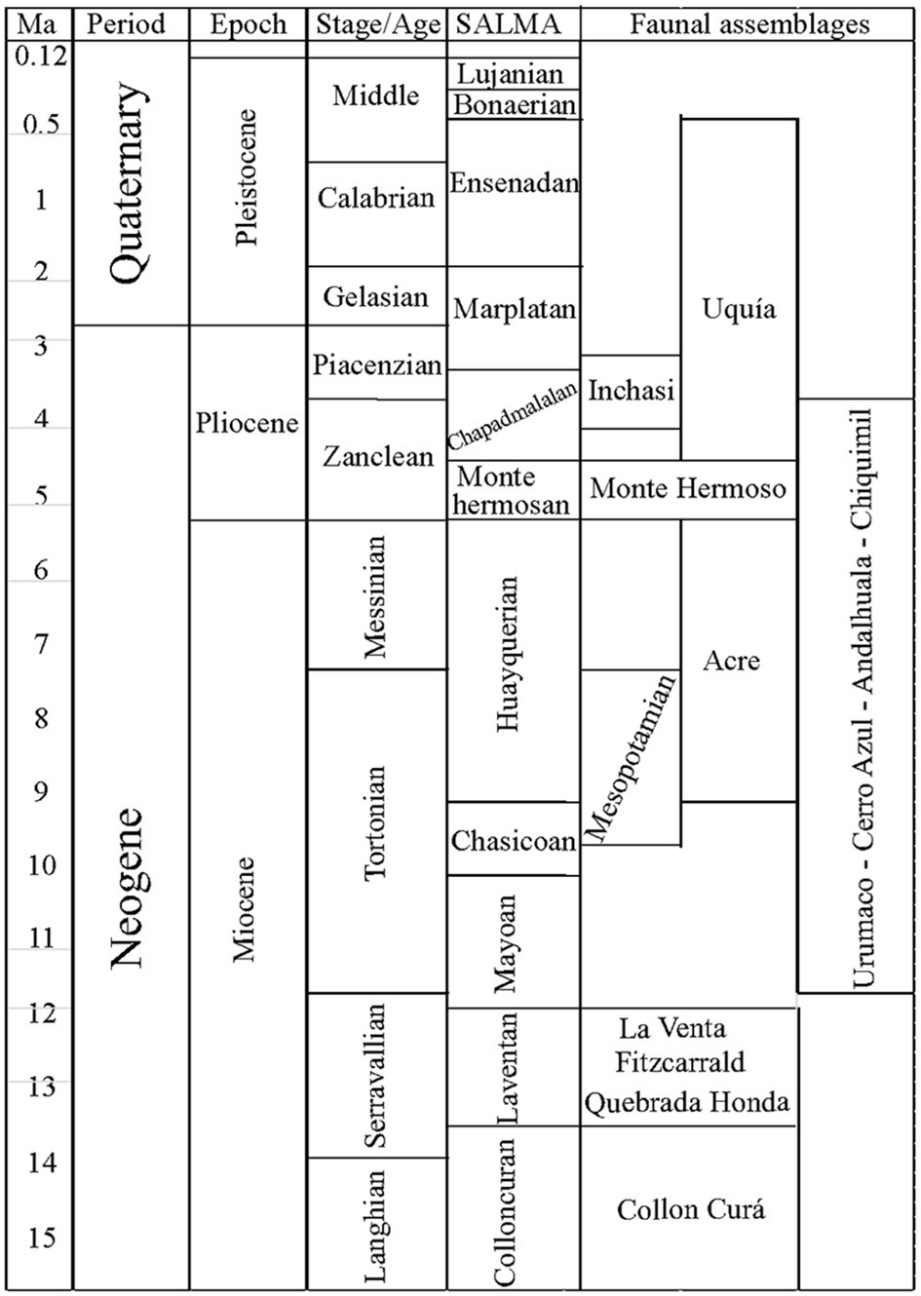
**Chronostratigraphy, South American Land Mammal Ages (SALMAs) and temporal distribution of the faunal assemblages discussed in the text**. Colloncuran:15.7–14 Ma (Madden et al., 1997) Laventan: 13.5–11.8 Ma. (Madden et al., 1997); Mayoan: 11.8–10 Ma. (Flynn and Swisher, 1995); Chasicoan: 10– ~8.5 (Flynn and Swisher, 1995); Huayquerian = ~8.5–5.28 Ma. Lower age following (Cione and Tonni, 2001; Reguero and Candela, 2011) and upper age following (Tomassini et al., 2013); Montehermosan = 5.28 –4.5/5.0 Ma. (Tomassini et al., 2013); Chapadmalalan = 4.5/5.0–3.3 (Tomassini et al., 2013); Marplatan = 3.3 – ~2.0 Ma. Lower age following (Tomassini et al., 2013) and upper age following (Cione and Tonni, 1999; Cione et al., 2007); Ensenadan = ~2.0–<0.78(0.5?) Ma. (Cione and Tonni, 1999; Cione et al., 2007); Bonaerian = <0.78(0.5?)–0.13 Ma. (Cione and Tonni, 1999); Lujanian = 0.13–0.08 Ma (Cione and Tonni, 1999).

Species lists from several middle and late Miocene–Pliocene mammal associations (La Venta, Fitzcarrald, Quebrada Honda, Collón Curá, Urumaco, Acre, Mesopotamian, Cerro Azul, Chiquimil, Andalhuala, Monte Hermoso, Inchasi and Uquía) were compiled from several sources (Goin et al., 2000; Cozzuol, 2006; Reguero and Candela, 2011; Brandoni, 2013; Tomassini et al., 2013; Tejada-Lara et al., in press) and other references available in the Paleobiology Database (PBDB) (Alroy, 2013), to which we added 450 references with records of Neogene fossil mammals from the Americas (Figures 2, 3; Supplementary Material 1–2). We obtained latitude and paleolatitude from each locality from the PBDB (Table 1) and estimated the distance in km among localities using Google Earth. Localities were coded for presence/absence at the generic level (Supplementary Table 1). The biochronology refers to the South American Land Mammal Ages (SALMA) and the calibration of the boundaries of Tomassini et al. (2013, modified from Cione et al., 2007) and Cione and Tonni (1999, 2001). Genera were used as taxonomic unit (including taxonomic identifications with *cf*. and *aff*. qualifiers). Lower taxonomical levels are still unresolved for several localities and data are incomparable.

**Table 1:** **Modern and ancient latitude and elevation of the faunal assemblages used in this study**.

**Faunal association**	**Latitude**	**Paleolatitude**	**Elevation**	**Paleoelevation**	**Biome**
La Venta	~3° N	~2.6° N	~380 m	“Lowland”	Tropical
Fitzcarrald	~10.5° S	~12° S	< 300 m	“Lowland”	Tropical
Quebrada Honda	~22° S	~22° S	~3500 m	~2600 ± 600 m	Temperate
Collón Curá	~40° S	~41° S	~800 m	?	Temperate
Urumaco	~11° N	~11° N	<100 m	“Lowland”	Tropical
Acre	~10° S	~10.5° S	<300 m	“Lowland”	Tropical
Mesopotamian	~32° S	~32° S	<100 m	“Lowland”	Temperate
Cerro Azul	~37° S	~37° S	~150 m	“Lowland”	Temperate
Chiquimil	~27° S	~27° S	1000–2500 m	?	Temperate
Andalhuala	~27° S	~27° S	1000–2500 m	?	Temperate
Monte Hermoso	~38° S	~38° S	<100 m	“Lowland”	Temperate
Inchasi	~19° S	~20° S	~3220 m	?	Temperate
Uquía	~23° S	~23° S	~2800 m	~1400–1700 m	Temperate

We analyzed closely contemporaneous fossil mammal associations from SA using the Bray-Curtis binary dissimilarity index. This reaches a maximum value of 1 when there are no shared taxa between the two compared communities. The Vegan package (Okasanen et al., 2013) was used to perform a cluster analysis with average grouping method and a Nonmetric Multidimensional Scaling (NMDS) set to two dimensions (axes) and 1000 runs. We compared tropical and temperate Miocene localities, and in order to account for differences in the sample size, we set the number of taxa equal to the assemblage with the lowest richness within the subgroup and calculate Bray-Curtis dissimilarity by resampling with replacement 1000 times all the localities. The Vegan package was used to obtain genera accumulation curves for tropical assemblages, using the random method. All analyses were performed in R (R Core Team, 2013).

We obtained records for late Miocene to late Pliocene land mammals for NA and SA from the PBDB. We classified each genus as North or South American if the taxon or its ancestor were in either NA or SA before 10 Ma. We compared the geographic distribution (tropical vs. temperate) and time of first appearance datum (FAD) of GABI migrants in the continent (Supplementary Material 3 and Supplementary Table 2). In order to account for the age uncertainty of each FAD, we generate 1000 different random values between the maximal and minimal age estimate and calculate the mean and standard deviation of the age estimate for each record.

### Study sites

We selected faunal associations from the tropical and temperate regions of South America which all together span from the middle Miocene (~15 Ma) to the late Pliocene (~2 Ma), a critical time period for the GABI. The study sites cover a wide latitudinal gradient across the continent (Table 1).

#### La venta

La Venta is one of the best-studied fossil assemblages from the Neotropics and among vertebrates includes freshwater fishes, crocodiles, turtles and different mammal clades (Kay et al., 1997). These come from the Honda Group in the central Magdalena valley, Colombia (Figure 2). Its age is constrained by radiometric and paleomagnetic data. The assemblage of La Venta served as the basis for defining the Laventan SALMA (middle Miocene, 13.5–11.8 Ma) (Madden et al., 1997).

#### Fitzcarrald

The localities of the Fitzcarrald assemblage are found along the Inuya and Mapuya rivers in the Amazon of Peru (Figure 2) from the Ipururo Formation, interpreted as middle Miocene (Laventan Age) (Antoine et al., 2007; Tejada-Lara et al., in press). The vertebrate assemblage includes fishes, turtles, crocodiles, snakes and 24 mammalian taxa (Negri et al., 2010; Tejada-Lara et al., in press).

#### Quebrada honda

Quebrada Honda is located in southern Bolivia at ~21°S latitude, 20 km north of the Argentine frontier and at an elevation of about 3500 m (Figure 2). The fossil-bearing deposits crop out in the valley of the Honda River and its tributaries. Paleomagnetic and radioisotopic data provide an extrapolated age of 13–12.7 Ma for the fossil bearing beds (MacFadden et al., 1990). Multiple proxies to estimate paleoelevation of the Central Andean Altiplano have yielded values between 1000 and 2000 m for the middle Miocene (Garzione et al., 2008); however, a most recent study using clumped isotope thermometry on paleosol carbonates inferred an earlier uplift for the Altiplano, with Quebrada Honda at about 2600 ± 600 m and a mean annual temperature of ~9 ± 5° C (Garzione et al., 2014). The assemblage includes about 30 mammals representing metatherians, xenarthrans, rodents, astrapotheres, litopterns and notoungulates and correspond to the Laventan SALMA (Croft, 2007).

#### Collón curá

The Collón Curá Formation is largely exposed at the west of Nord-Patagonian Massif (Neuquén and Río Negro provinces, and Norwest Chubut Province). The rich vertebrate association is represented by reptiles, birds, and principally mammals: metatherians, xenarthrans, rodents, notoungulates, litopterns, and astrapotheres (Kramarz et al., 2011). The fossil mammals collected in the vicinities of the Collón Curá river by Santiago Roth in the late 19th Century are the basis for the definition of the Colloncuran SALMA, although a critical review of most of the findings is still pending. Several radiometric dates for the Collón Curá Formation indicate ages between 15.5 and 10 Ma for the vertebrate association (e.g., Rabassa, 1974, 1978; Marshall et al., 1977; Bondesio et al., 1980; Mazzoni and Benvenuto, 1990; Madden et al., 1997).

#### Urumaco

The Urumaco sequence is found in the Falcón State in northwestern Venezuela (Figure 2). It includes the Querales, Socorro, Urumaco, Codore and San Gregorio formations, which together span from the middle Miocene to late Pliocene (Quiroz and Jaramillo, 2010). The Urumaco sequence shows a high diversity of crocodilians (Scheyer et al., 2013) and xenarthrans (Carlini et al., 2006a,b, 2008a,c). We focus our analysis on the Urumaco Formation. Linares (2004), on the basis of a mammal list of undescribed material suggested a middle to late Miocene age. Until a detail taxonomic revision is conducted, the biostratigraphic correlation of the Urumaco association remains tentative.

#### Acre

The Acre region in the southwestern Amazonia includes several fossiliferous localities which would represent different time intervals considering the geological and palinological evidence (Cozzuol, 2006). Fossil vertebrates come from the Solimões Formation of the state of Acre, Brazil and Peruvian and Bolivian localities from the Madre de Dios Formation (Negri et al., 2010) (Figure 2). The vertebrate assemblage is very diverse and includes fishes, snakes, lizards, birds, turtles, crocodiles, and mammals including whales, dolphins, manatees and a diverse assemblage of terrestrial forms. The Acre mammal assemblage has been referred to late Miocene, Huayquerian SALMA (Cozzuol, 2006; Ribeiro et al., 2013) or included also in the Pliocene, Montehermosan SALMA (Cozzuol, 2006). Campbell et al. (2001) reported ^40^A/^39^A dates of 9.01 ± 0.28 Ma for the base of the Madre de Dios Formation and 3.12 ± 0.02 Ma near the top.

#### Mesopotamian

The continental mammals of the Mesopotamian assemblage come from the lower levels of the Ituzaingó Formation, which crops out along the cliffs of the Paraná River in Corrientes and Entre Ríos provinces, north-east Argentina (Figure 2). The vertebrate assemblage is rich and includes fishes, crocodiles, birds and mammals (Cione et al., 2000; Brandoni and Noriega, 2013). It differs taxonomically from other associations in Argentina at the same latitudes and this was explained by a southern extension of the northern realm (Cozzuol, 2006). The age of the Mesopotamian assemblage has been largely debated (Cione et al., 2000 and references therein); it is currently assigned to the late Miocene, Huayquerian SALMA (Cione et al., 2000) or also extended into the Chasicoan SALMA (Brandoni, 2013; Brunetto et al., 2013). The dating of 9.47 Ma for the upper levels of the lower Paraná Formation (Pérez, 2013) represents a maximum limit for the Mesopotamian assemblage.

#### Cerro azul

Several localities in central east Argentina (La Pampa and Buenos Aires provinces) have provided abundant fossil vertebrates from the Cerro Azul and Epecuén formations which are considered geologically correlated (Goin et al., 2000). This assemblage includes reptiles, birds and a rich mammal association. These units are assigned to the late Miocene, Huayquerian SALMA (Goin et al., 2000; Montalvo et al., 2008; Verzi and Montalvo, 2008; Verzi et al., 2011) on the basis of mammal biostratigraphy. This association is currently the most complete list for this age (Goin et al., 2000). The possibility of extension into the late Pliocene cannot be discarded for some localities assigned to the Cerro Azul Formation (Prevosti and Pardiñas, 2009).

#### Chiquimil

The Chiquimil Formation is exposed in north-west Argentina (Catamarca Province) and is divided in three members. The Chiquimil A (Riggs and Patterson, 1939; Marshall and Patterson, 1981) or El Jarillal Member (Herbst et al., 2000; Reguero and Candela, 2011) provided a rich fossil record. The mammalian association has been assigned to the late Miocene, Huayquerian SALMA (Reguero and Candela, 2011). A dating in the middle section of the Chiquimil Formation indicated ~6.68 Ma (Marshall and Patterson, 1981).

#### Andalhuala

The Andalhuala Formation is exposed in the Santa María Valley in north-west Argentina (Catamarca Province). This is a classical fossiliferous unit of the South American Neogene with abundant and diverse fossil remains, including plants, invertebrates, and vertebrates (Riggs and Patterson, 1939; Marshall and Patterson, 1981). Basal levels of the Andalhuala Formation have been dated to ~7.14 Ma (Latorre et al., 1997) and ~6.02 Ma (Marshall and Patterson, 1981) while a tuff sample close to the upper part of the sequence was dated to ~3.53 Ma (Bossi et al., 1993). The mammal association has been referred to the Montehermosan–Chapadmalalan SALMAs (Reguero and Candela, 2011).

#### Monte hermoso

The Monte Hermoso Formation is exposed in the Atlantic coast at the south west of Buenos Aires Province, Argentina. This unit has provided fishes, anurans, reptiles, birds, and a diverse mammal association. Recent biostratigraphic and biochronological analyses (Tomassini and Montalvo, 2013; Tomassini et al., 2013) have recognized a single biozone (the *Eumysops laeviplicatus* Range Zone) in the Montehermosan Formation which is the base for the Montehermosan SALMA. The Montehermosan was restricted to the early Pliocene between <5.28 and 4.5/5.0 Ma by considering the dating of 5.28 Ma in levels with Huayquerian mammals and paleomagnetic correlations in the upper Chapadmalal Formation (Tomassini et al., 2013).

#### Inchasi

The locality of Inchasi is found in the eastern cordillera in the department of Potosí, Bolivia at an elevation of about 3220 masl and ~19°S latitude (Figure 2). The mammal assemblage includes 10 mammals, representing xenarthra, rodentia, and native ungulates (Litopterna and Notoungulata) (Anaya and MacFadden, 1995). Paleomagnetic analysis indicates an age of about 4–3.3 Ma. The analysis of the mammal association first suggested Montehermosan and/or Chapadmalalan ages (MacFadden et al., 1993). A later revision (Cione and Tonni, 1996) correlated Inchasi with the Chapadmalalan, although probably older than the classical Chapalmalalan sections at the Atlantic coast.

#### Uquía

The Uquía Formation crops out in the Quebrada de Humahuaca, Jujuy province, north western Argentina at an elevation of ~2800 masl and ~23°S latitude (Figure 2). The Uquía Formation is divided in three units: the Lower Unit was assigned to the late Chapadmalalan, the Middle Unit to the Marplatan (Vorhuean, Sanandresian), and the Upper Unit to the Ensenadan (Reguero et al., 2007; Reguero and Candela, 2011). ^40^K–^40^Ar data from a volcanic tuff (“Dacitic tuff”) in the Lower Unit provided ~3.0 Ma. Another tuff (U1) dated as 2.5 Ma is the boundary between the Middle and Upper Unit. The geological and paleontological evidence suggested that during the late Pliocene the area was a wide intermountain valley at about 1700–1400 masl (Reguero et al., 2007).

## Results

### Middle and late miocene–pliocene mammal faunas from SA

**Figure 4:**
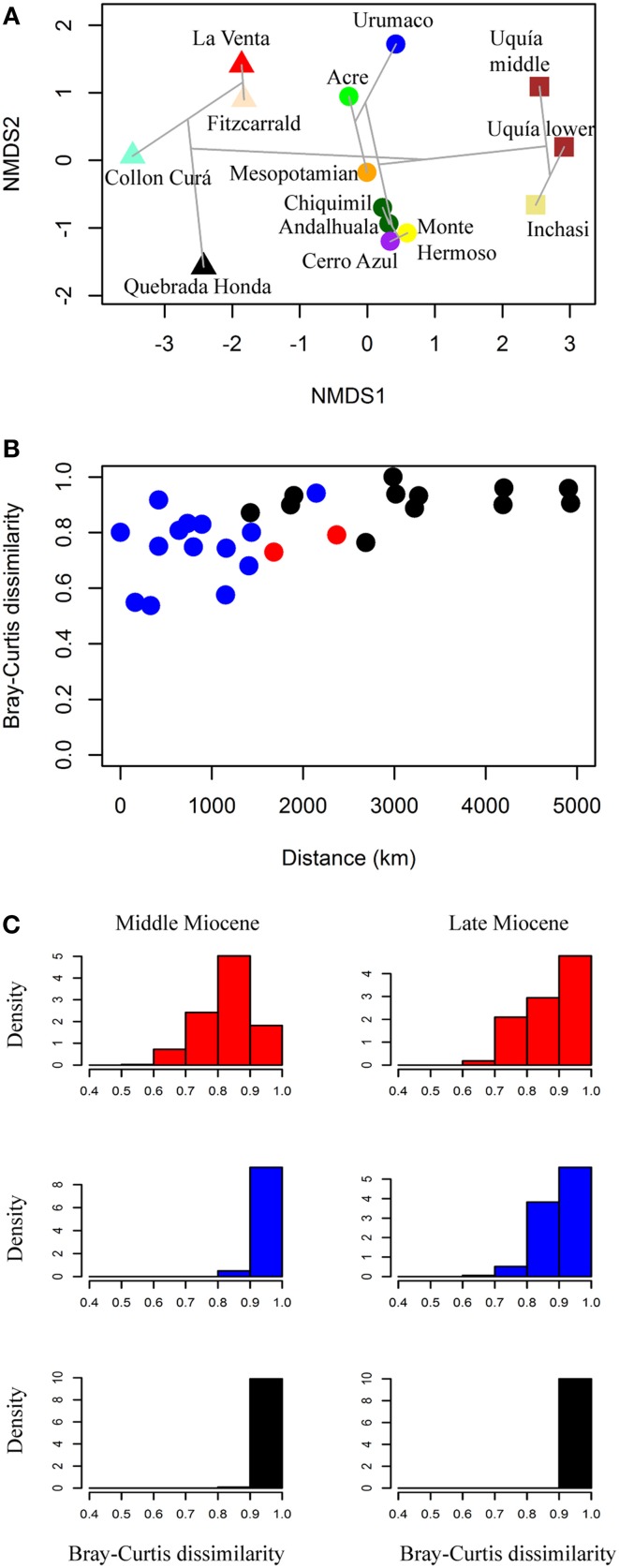
**(A)** NMDS plot of the faunal associations using Bray-Curtis dissimilarity; triangles, middle Miocene; circles, late Miocene; squares, Pliocene. The gray lines show the clustering result. **(B)** Bray-Curtis dissimilarity relationship with distance in km, between each locality pair. We include only localities pairs which are within the same time interval (middle Miocene, late Miocene, Pliocene), red, tropical–tropical pair; blue, temperate–temperate pair; black, tropical–temperate pair. **(C)** Density histograms of the Bray-Curtis dissimilarity values among the different faunal associations analyzed for the middle and late Miocene, red, only tropical faunas, blue, only temperate faunas, black, tropical vs. temperate faunas.

In the NMDS analysis (stress value = 0.083), the analyzed South American localities are primarily grouped by age and secondarily by geographic position (Figure 4A). The NMDS1 clearly separates middle Miocene, late Miocene and Pliocene localities and for the middle and late Miocene assemblages, the NMDS2 separates tropical from temperate localities. For the middle Miocene (Colloncuran, Laventan), the cluster analysis separates the tropical assemblages of La Venta (~2.6°N paleolatitude) and Fitzcarrald (~12.5°S paleolatitude) from the southern Collón Curá (~41.3°S paleolatitude) and Quebrada Honda (~22.3°S paleolatitude). For the late Miocene (Huayquerian–Montehermosan), Urumaco (~10.9°N paleolatitude) appears outside the groups formed by Acre (~10.5°S paleolatitude) and Mesopotamian (~32.5°S paleolatitude), another cluster includes the Argentinean assemblages of Andalhuala (~26.8°S paleolatitude), Chiquimil (~27.0°S paleolatitude), Cerro Azul (~37.0°S paleolatitude), and Monte Hermoso (~38.9°S paleolatitude). Finally, the early Pliocene (Chapadmalalan– Marplatan) temperate associations from Inchasi (~19.9°S paleolatitude) and Uquía (~23.4°S paleolatitude) cluster together, although there are no tropical assemblages to compare with. If we compare only faunal assemblages from the same time period (middle Miocene, late Miocene and Pliocene), there is a positive relationship between the Bray-Curtis dissimilarity and the distance of each pair of assemblages studied (Figure 4B).

The Bray-Curtis dissimilarity values with resampling calculated for the tropical, temperate and tropical vs. temperate assemblages for the middle and late Miocene shows that all the assemblages are very different (Figure 4C). The Bray-Curtis dissimilarity between middle Miocene tropical (La Venta and Fitzcarrald) and temperate (Quebrada Honda and Collón Curá) assemblages compared to the dissimilarity between tropical vs. temperate are found to be statistically significant. Dissimilarity values of middle Miocene tropical (mean = 0.830) are lower than middle Miocene tropical vs. temperate (mean = 0.956) (Mann-Whitney U, *p* < 2.2 e-16); whereas middle Miocene temperate dissimilarity (mean = 0.964) is higher than middle Miocene tropical vs. temperate dissimilarity (Mann-Whitney U, *p* ≤ 2.87 e-15). For the late Miocene, dissimilarity of tropical assemblages (Acre and Urumaco) is lower (mean = 0.873) than tropical vs. temperate (mean = 0.969) (Mann-Whitney U, *p* < 2.2 e-16). We also found difference between temperate assemblages (Mesopotamian, Chiquimil, Andalhuala, Cerro Azul, and Monte Hermoso; mean = 0.899) and tropical vs. temperate dissimilarity (Mann Whitney U, *p* < 2.2e-16).

**Figure 5:**
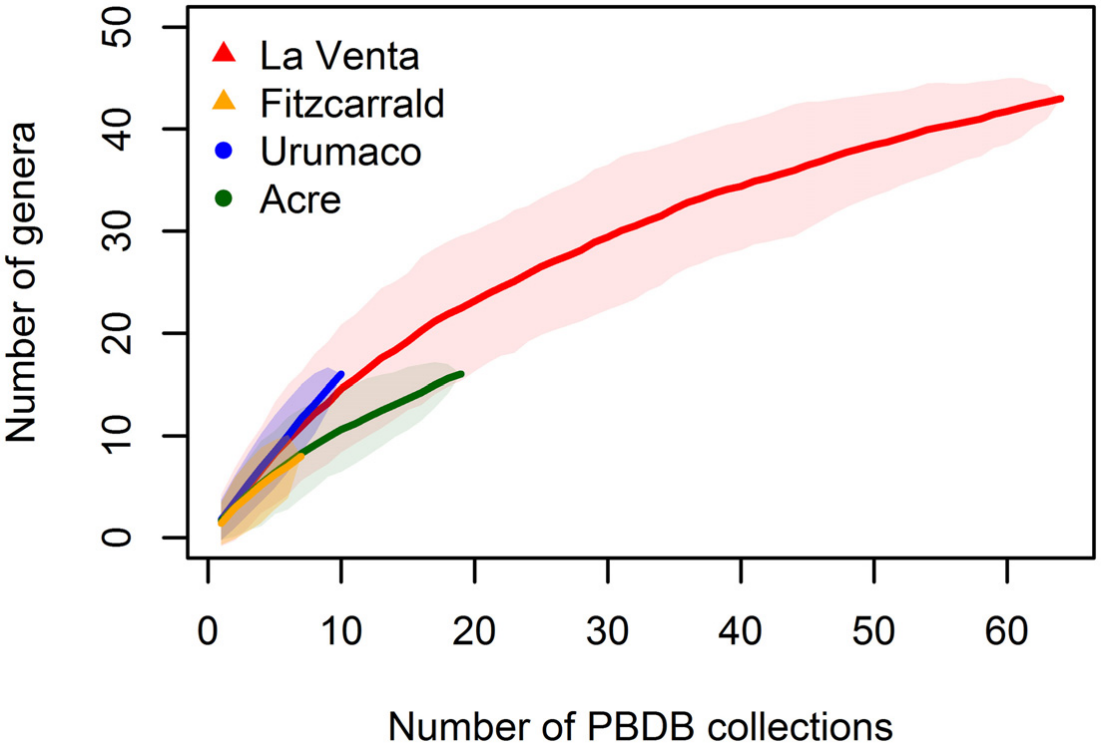
**Accumulation curves estimated with random method for the tropical faunal associations, shaded areas represent the 95% confidence interval**.

The number of PBDB collections was used to generate accumulation curves for the tropical assemblage (Figure 5). Each collection represents a geographic and stratigraphic point where the fossils have been found and provide a good proxy for sampling effort. We excluded from the analysis the Acre collection with unknown stratigraphic provenance. The accumulation curves show that generic richness for tropical assemblages is underestimated, even for the better known assemblage of La Venta.

### Temporal and spatial distribution patterns of GABI

**Figure 6:**
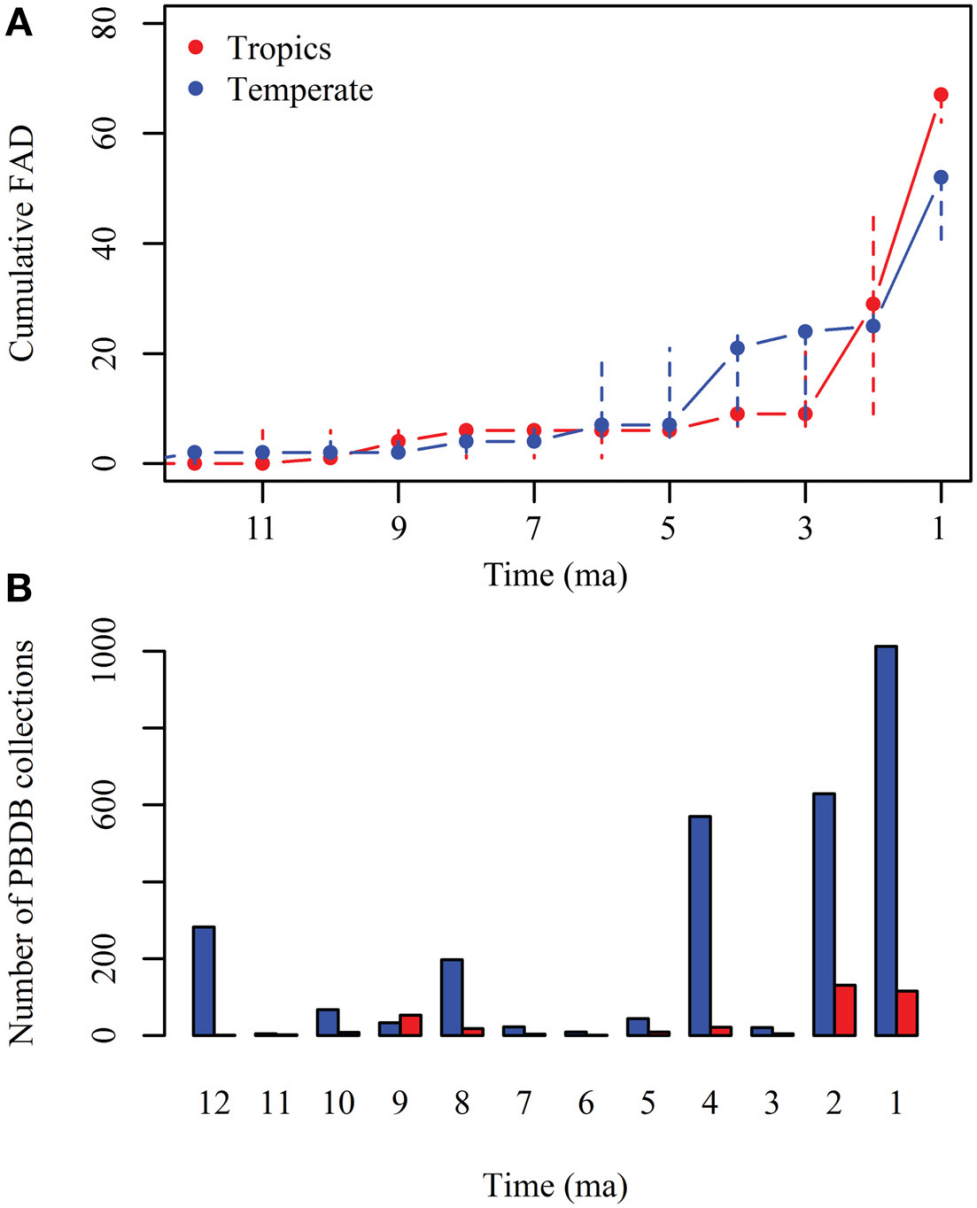
**(A)** Cumulative first appearance datum (FAD) of GABI participants in North and South America for each million year since 12 Ma; red, FADs record in the tropics; blue, FADs record in the temperate regions. Solid circles represent the mean and dashed lines the standard deviation. **(B)** Number of collections with records of land mammals in the Paleobiology Database (PBDB) for each million year since 12 Ma; red, collections in the tropics; blue= collections in the temperate region.

The cumulative first appearance datum (FAD) of non-native taxa for both NA and SA continents (Figure 6A, Supplementary Table 2) shows that first migrations are recorded in the temperate region (cumulative FAD mean = 2 by 10 Ma), represented by the ground sloths *Thinobadistes* (Mylodontidae) and *Pliometanastes* (Megalonychidae) recorded at McGehee Farm, Florida (Hirschfeld and Webb, 1968; Webb, 1989). During the late Miocene (12–5 Ma), the number of FAD is similar between the tropics (cumulative FAD mean = 6 by 5 Ma) and temperate (cumulative FAD mean = 7 by 5 Ma). In the tropics, the oldest records of migrants are those from the Acre region in Peru (Campbell et al., 2010; Prothero et al., 2014) of disputable age (Alberdi et al., 2004; Lucas and Alvarado, 2010; Lucas, 2013). During the Pliocene (between 3 and 4 Ma) there is an increase in the number of FAD at higher latitudes (cumulative FAD mean = 21), but this is not recorded in the tropics (cumulative FAD mean = 9). Finally, during the Pleistocene (2–1 Ma) a higher number of FADs are recorded in tropical and temperate regions. Most of the collections in the PBDB with records of land mammals in the Americas are in the temperate region and are younger than 4 Ma (Figure 6B).

## Discussion

### Middle and late miocene–pliocene mammal faunas from SA

The NMDS1 shows that a strong temporal component establishes the dissimilarity relationships among the faunas. In addition, an important influence of the geographic position is reflected in the distribution of the faunas along the NMDS2 axis. There is a positive relationship between the Bray-Curtis dissimilarity values and the distance between faunas (Figures 4 A,B).

For the middle Miocene, Colloncuran–Laventan faunal associations, a differentiation between the tropical assemblages of La Venta and Fitzcarrald, and the southern Quebrada Honda and Collón Curá was observed (Figure 4A). The middle latitude fauna Quebrada Honda appears unique, although it is closer to the slightly older and temperate Collón Curá than to the contemporaneous tropical faunas of La Venta and Fitzcarrald (Croft, 2007; Tejada-Lara et al., in press). The reconstructed paleoenvironment for the middle Miocene Monkey Beds assemblage at La Venta considered an estimated annual rainfall between 1500 and 2000 mm using diet, locomotion and body size indices of the mammal community (Kay and Madden, 1997a,b).

For the late Miocene assemblages, the NMDS indicates a high dissimilarity between the tropical faunas of Urumaco and Acre. For the Urumaco mammal assemblage, xenarthrans and rodents are the most conspicuous elements, but further studies on other clades promise to document a higher diversity than currently recognized. The temperate assemblages of Chiquimil, Andalhuala, Cerro Azul, and Monte Hermoso cluster together and the Mesopotamian is between this group and Acre (Figure 4A).

After taking into account the differences in sample size, we found that the dissimilarity values of tropical assemblages (mean = 0.830 for middle Miocene, and mean = 0.879 for late Miocene) and late Miocene temperate assemblages (mean = 0.899 for late Miocene) are lower than the values for tropical vs. temperate assemblages (mean = 0.956 for middle Miocene and mean = 0.969 for late Miocene) (Figure 4C). Consequently, the Bray-Curtis dissimilarity between faunas of the same age and biome is lower than between faunas of different biomes (tropical vs. temperate); although, the mean dissimilarity values in all cases are high (>0.8).

As shown by the accumulation curves (Figure 5), the generic richness of the tropical assemblages studied are underestimated. A more comprehensive knowledge of tropical faunas is needed to better understand the paleodiversity patterns and paleobiogeography in the new world.

### Temporal and spatial distribution patterns of GABI

The cumulative FAD across time of GABI participants in each continent shows that the GABI was a gradual process that began in the late Miocene (~10 ma) (Figure 6A). The early phase of GABI (pre GABI *sensu* Woodburne, 2010) is characterized by a small number of migrants, with a mean cumulative FAD = 6 between 4 and 5 Ma in the tropics and a cumulative FAD = 7 in the temperate region. The land connection between the two continents occurred at the Isthmus of Panama, located within the tropical zone. Therefore, it would be expected that the Neotropics record the earliest GABI immigrants, but older immigrants have been found at higher latitudes.

The findings reported by Campbell and colleagues (Campbell et al., 2010; Frailey and Campbell, 2012; Prothero et al., 2014) in the Acre region of the Amazon basin, assigned to late Miocene (~9 Ma) sediments would represent the oldest NA immigrants. However, the dromomerycine artiodactyl, peccaries, tapirs, and gomphotheres have not been found in other late Miocene localities in SA and these findings await further clarifications. In SA, the most frequent pre-GABI elements are procyonids recorded in several late Miocene–Pliocene (Huayquerian–Chapadmalalan) SA localities since ~7.3 Ma (Cione et al., 2007; Reguero and Candela, 2011; Forasiepi et al., 2014). The evidence of the fossil record combined with the living species distribution suggests that much of the evolutionary history of procyonids occurred in the Neotropics, possibly in SA (Eizirik, 2012). Molecular studies have predicted that the diversification of the group occurred in the early Miocene (~20 Ma), with most of the major genus-level lineages occurring in the Miocene (Koepfli et al., 2007; Eizirik et al., 2010; Eizirik, 2012). This scenario requires a bias in the fossil record, claims an evolutionary history for procyonids in SA that largely precedes the GABI, and suggests an arrival into SA long before previously thought as for several other mammalian clades (Almendra and Rogers, 2012; and references therein).

Since 4 Ma, the number of FAD at higher latitudes rapidly increases and this trend continues during the Pleistocene. In contrast, the number of FAD in the tropics remains low during the Pliocene (cumulative FAD mean = 9 by 2–3 Ma), but rapidly increases during the Pleistocene. A large difference in the number of PBDB collections across time and latitude is observed for land mammals for the last 12 Ma (Figure 6B). Most records come from higher latitudes and are younger than 4 Ma, by the time the FAD increases; this suggest that temporal and geographic patterns of GABI are influenced by the sampling bias toward high latitudes and the higher number of Pliocene–Pleistocene records.

The migration of northern taxa into SA after the completion of the land bridge by ~3 Ma was correlated with supposed expansion of savannas and grasslands in the Neotropics during glacial periods (Webb, 1991, 2006; Leigh et al., 2014). The expansion of savannas during glacial times has been questioned (Behling et al., 2010). If this is the case, the Andes could have served as route of migration of northern taxa toward temperate environments in SA (Webb, 1991), as NA taxa seem to have been more successful in temperate biomes whereas SA taxa dominate in the tropics (Webb, 1991, 2006; Leigh et al., 2014).

## Conclusions

The dissimilarity analysis primarily grouped the faunal assemblages by age and secondarily by geographic distribution. The dissimilarity values among the fossil faunal assemblages analyzed support the differentiation between tropical and temperate assemblages in SA during the middle Miocene (Colloncuran–Laventan) and late Miocene (Huayquerian–Montehermosan). The mid-latitude, middle Miocene assemblage of Quebrada Honda has higher affinities with the slightly older and temperate Collon Curá than with the tropical assemblages of La Venta and Fitzcarrald. For the late Miocene, the temperate assemblages of Chiquimil, Andalhuala, Cerro Azul, and Monte Hermoso cluster together, while the Mesopotamian is between this group and the tropical assemblages of Acre and Urumaco.

The cumulative FAD across time and latitude shows that faunisitc movements related to GABI began during the late Miocene (~10 Ma) with the oldest records found at higher latitudes. The number of FAD remained relatively low until 4–5 Ma when FAD starts to increase, peaking during the Pleistocene.

The study of paleodiversity patterns and paleobiogeography in the Americas is challenged by the sampling bias toward higher latitudes and the still scarce data from tropical faunas. The interpretation of the temporal and geographic patterns of GABI is likely influenced by these sampling issues.

## Author contributions

Conceived and designed: Juan D. Carrillo, Analía Forasiepi, Carlos Jaramillo, Marcelo R. Sánchez-Villagra. Compiled bibliographic data: Juan D. Carrillo, Analía Forasiepi F, Carlos Jaramillo. Analyzed data: Juan D. Carrillo, Carlos Jaramillo. Wrote the paper: Juan D. Carrillo, Analía Forasiepi. All authors contributed to the final interpretation and editing of the manuscript.

### Conflict of interest statement

The authors declare that the research was conducted in the absence of any commercial or financial relationships that could be construed as a potential conflict of interest.
