# Supplementary material for: Neotropical mammal diversity and the Great American Biotic Interchange: spatial and temporal variation in South America's fossil record
Source: Front Genet. 2015 Jan 5;5:451. doi: 10.3389/fgene.2014.00451 (PMC4283609; doi:10.3389/fgene.2014.00451)
Supplement: Supplementary file 3 [file DataSheet1.DOCX]

***Supplementary Material***

**Neotropical mammal diversity and the Great American Biotic Interchange: spatial and temporal variation in South America’s fossil record**

**Juan D. Carrillo^1,3^*, Analía Forasiepi^2^, Carlos Jaramillo^3^ , Marcelo R. Sánchez-Villagra^1^**

^1^ Paläontologisches Institut und Museum, Universität Zürich, Zurich, Switzerland

^2^ IANIGLA, CCT-CONICET Mendoza, Av. Ruiz Leal s/n, 5500 Mendoza, Argentina

^3^ Smithsonian Tropical Research Institute, Panama, Panama

*** Correspondence:** Juan D. Carrillo, Paläontologisches Institut und Museum, Universität Zürich , Karl-Schmid-Strasse 4 8006, Zurich,Switzerland.

Juan.carrillo@pim.uzh.ch

1. **Supplementary Material 1**

Taxonomic lists of the mammal faunal assemblages analyzed

**La Venta:** *Anadasypus, Aotus, Boreostemma, Eodolichotis, Eumops, Glossotheriopsis, Granastrapotherium, Hondathentes, Huilatherium, Kiotomops, Lagonimico, Marmosa, Micodon, Microscleromys, Microsteiromys, Miocallicebus, Nanoastegotherium, Neoglyptatelus, Neonematherium, Neosaimiri, Noctilio, Notonycteris, Nuciruptor, Olenopsis, Pachybiotherium, Palynephyllum, Patasola, Pedrolypeutes, Pericotoxodon, Potamops, Prodolichotis, Prolicaphrium, Pseudoprepotherium, Rhodanodolichotis, Ricardomys, Scirrotherium, Scleromys, Stirtonia, Theosodon, Thylamys, Thyroptera, Xenastrapotherium.*

**Fitzcarrald:** *Acarechimys, Boreostemma, Drytomomys, Granastrapotherium, Megathericulus, Miocochilius, Neoepiblema, Neoglyptatelus, Parapropalaehoplophorus, Pericotoxodon, Potamarchus, Prodolichotis, Scleromys, Tetramerorhinus, Theosodon, Urumacotherium, Xenastrapotherium.*

**Quebrada Honda:** *Acarechimys, Acyon, Guiomys, Hapalops, Hemihegetotherium, Hiskatherium, Hondalagus, Mesoprocta, Miocochilius, Paratrigodon, Plesiotypotherium, Prolagostomus, Prozaedyus, Quebradahondomys.*

**Collón Curá:** *Abderites, Acarechimys, Acdestis, Alloiomys, Amphibradys, Anisolophus, Arctodictis, Branisamyopsis, Diellipsodon, Eocardia, Eonaucum, Epipatriarchus, Eucholaeops, Eucinepeltus, Eutrachytherus, Galileomys, Glossoptheriopsis, Hegetotherium, Homalodotherium, Hyperoxotodon, Icochilus, Interatherium, Maruchito, Massoiamys, Megastus, Megathericulus, Neoreomys, Neosteiromys, Nesciotherium, Nesodon, Pachyrukhos, Palyeidodon, Patagosmilus, Pitheculites, Planops, Pliolagostomus, Prepotherium, Proeutatus, Prolagostomus, Propithecia, Protacaremys, Prothylacynus, Prototrigodon, Protypotherium, Prozaedius, Pseudonotictis , Pseuhapalops, Scleromys, Steiromys, Stenotatus, Stereotoxodon, Stilotherium, Theosodon, Vetelia.*

**Urumaco:** *Bolivartherium, Bounodus, Cardiatherium, Eumegamys, Gyrinodon, Lestodon, Mirandabradys, Ocnerotherium, Olenopsis, Phoberomys, Potamarchus, Tetrastylus, Urumacotherium, Urumaquia.*

**Acre:** *Abothrodon, Acrecebus, Amahuacatherium, Anadasypus, Asterostemma, Cardiatherium, Cullinia, Didelphis, Eumegamys, Gyriabrus, Gyrinodon, Hapalops, Kraglievichia, Lutreolina, Mesenodon, Mesotoxodon, Minitoxodon, Neoepiblema, Neoglyptatelus, Neotoxodon, Neotrigodon, Noctilio, Octodontobradys, Palaeotoxodon, Paraglyptodon, Paratrigodon, Phoberomys, Planops, Plesiotoxodon, Pliomorphus, Plohophorus, Potamarchus, Proterotherium, Protomegalonyx, Pseudoprepotherium, Purperia, Ranculcus, Scirrotherium, Simplimus, Solimoea, Stenodon, Stirtonia, Surameryx, Sylvochoerus, Telicomys, Tetrastylus, Toxodontherium, Trigodon, Trigodonops, Urumacotherium, Waldochoerus, Xenastrapotherium, Scleromys.*

**Mesopotamian:** *Amphiocnus, Berthawyleria, Borhyaena, Brachytherium, Cardiatherium, Cardiomys, Carlesia, Caviodon, Chasicotatus, Chironectes, Chlamyphractus, Comaphorus, Cullinia, Cyonasua, Dasypus, Diadiaphorus, Dilobodon, Dinotoxodon, Drytomomys, Eleutherocercus, Eomegatherium, Eumegamys, Eumegamysops, Eumysops, Eutemnodus, Eutomodus, Eutypotherium, Gyriabrus, Haplodontherium, Haplostropha, Hoplophorus, Isostylomys, Kraglievichia, Lagostomus, Macroeuphractus, Megabradys, Megalonychops, Munizia, Myocastor, Neobrachytherium, Neoepiblema, Neohapalops, Notictis, Octomylodon, Ortotherium, Oxyodontherium, Pachynodon, Palaeocavia, Palaeohoplophorus, Palaeotoxodon, Paradoxomys, Parahoplophorus, Paranabradys, Paranamys, Paranauchenia, Parodimys, Philander, Phoberomys, Pliodolichotis, Pliomegatherium, Pliomorphus, Plohophorus, Potamarchus, Prodolichotis, Proeuphractus, Promacrauchenia, Promegatherium, Promylodon, Pronothrotherium, Protabrocoma, Proterotherium, Protoglyptodon, Protomegalonyx, Protypotherium, Pseudoeuryurus, Pyramiodontherium, Ranculcus, Scalabrinitherium, Scirrotherium, Sphenotherus, Stenotephanos, Strabosodon, Strophostephanos, Stylocynus, Tetrastylus, Thylacosmilus, Torcellia, Toxodontherium, Urotherium, Xotodon, Zygolestes.*

**Cerro Azul:** *Aspidocalyptus, Borhyaenidium, Cardiomys, Chasichimys, Chasicotatus, Chorobates, Clyomys, Coscinocercus, Cyonasua, Doellotatus, Elassotherium, Eoauchenia, Eosclerocalyptus, Epecuenia, Eumysops, Hemihegetotherium, Hyperdidelphys, Lagostomus, Lutreolina, Macrauchenia, Macrochorobates, Macroeuphractus, Microtragulus, Neophanomys, Orthomyctera, Paedotherium, Palaeocavia, Palaeoctodon, Paleuphractus, Phtoramys, Pisanodon, Pithanotomys, Plesiomegatherium, Pliolestes, Plohophorus, Proeuphractus, Promacrauchenia, Pseudotypotherium, Tetrastylus, Thylacosmilus, Thylamys, Thylatheridium, Xenodontomys, Zygolestes.*

**Chiquimil:** *Cardiomys, Chasicotatus, Chorobates, Diadiaphorus, Eosclerocalyptus, Gyriabrus, Lagostomopsis, Macrochorobates, Nephanomys, Orthomyctera, Paedotherium, Paleuphractus, Paraeuphractus, Paranamys, Potamarchus, Proscelidodon, Protabrocoma, Pseudohegetotherium, Stromaphorus, Tetrastylus, Toxodontherium, Tremacyllus, Typotheriopsis, Vassallia, Vetelia, Xotodon.*

**Andalhuala:** *Cardiomys, Chapalmalania, Chasicotatus, Chorobates, Cyonasua, Eosclerocalyptus, Glyptodontidium, Hemihegetotherium, Hesperocynus, Hyperdidelphys, Lagostomopsis, Lutreolina, Macrochorobates, Macroeucphractus, Microtragulus, Neobrachytherium, Neophanomys, Orthomyctera, Palaeocavia, Paraeuphractus, Phlyctaenopyga, Pithanotomys, Prodolichotis, Promacrauchenia, Pronothrotherium, Protabrocoma, Pyramiodontherium, Sphenotherus, Stromaphorus, Tetrastylus, Thylacosmilus, Toxodontherium, Treamcyllus, Typotheriopsis, Urotherium, Xotodon.*

**Monte Hermoso:** *Actenomys, Alitoxodon, Argyrolagus, Auliscomys, Cardiomys, Caviodon, Chorobates, Cyonasua, Diheterocnus, Diplasiotherium, Doellotatus, Eleutherocercus, Eoauchenia, Eosclerocalyptus, Epitherium, Eucelophorus, Eumysops, Holozaedyus, Hyperdidelphys, Lagostomus, Lestodon, Lutreolina, Macrochorobates, Macroeuphractus, Microcavia, Microtragulus, Myrmecophaga, Necromys, Neocavia, Neopachtus, Neophanomys, Notocynus, Orthomyctera, Paedotherium, Palaeocavia, Palaeodaedicurus, Pampatherium, Parahyaenodon, Paramyocastor, Phtoramys, Phugatherium, Phyctaenopyga, Pithanotomys, Plaina, Plohophoroides, Plohophorus, Prodolichotis, Promacrauchenia, Proscelidodon, Pseudoplataeomys, Pseudotypotherium, Reithrodon, Ringueletia, Sparassocynus, Telicomys, Thylacosmilus, Thylamys, Thylaohorops, Thylatheridium, Toxodon, Trachycalyptus, Tremacyllus, Trigodon, Xotodon.*

**Inchasi:** *Caviodon, Glossotherium, Hypsitherium, Paraglyptodon, Phugatherium, Plohophorus, Posnanskytherium, Promacrauchenia, Proscelidodon, Vassallia.*

**Uquía:** Lower: *Ctenomys, Doellotatus, Microcavia, Paraglyptodon, Scelidotheridium, Vassallia;* Middle: *Chaetophractus, Ctenomys, Erethizon, Hippidion, Hydrochoeropsis, Lestodon, Megatherium, Panochthus, Paraglyptodon, Platygonus, Pyramiodontherium, Urotherium, Windhausenia, Xiphuroides.*

1. **Supplementary Material 2**

Search queries used and Paleobiology Database (PBDB) ID’s of the collections belonging to the different mammal assemblages used in the analysis.

**Queries:**

Taxonomic level: genus

Taxon or taxa to include: Mammalia

Taxon or taxa to exclude: Cetacea, Sirenia

**La Venta**

Date of download: 13.04.2014

PBDB collection ID’s: 13630, 13650, 13653, 13678, 13680, 13681, 13682, 13684, 13685, 13686, 13687, 13739, 13740, 13741, 13743, 13747, 13749, 13750, 13754, 13757, 13758, 94555, 117981, 132559, 132580, 133441, 133648, 133688, 133689, 133692, 133693, 133780, 134806, 135348, 135373, 136211, 136233, 139894, 140007, 140012, 140046, 140051, 140179, 140181, 140183, 140634, 141256, 141257, 142428, 142486, 142494, 143489, 143779, 143781, 143785, 143787, 143788, 143789, 143791, 143795, 144401, 145175, 145191, 145194, 145234, 145432, 145559, 145614, 145617, 146527

**Fitzcarrald**

Date of download: 19.11.2014

PBDB collection ID’s: 107992, 107993, 144792, 144793, 144794, 144795, 144950,163839,163840,163841, 163842, 163843, 163844

**Quebrada Honda**

Date of download: 13.04.2014

PBDB collection ID’s: 38071, 133664, 133629

**Collón Curá**

Date of download: 04.07.2014

PBDB collection ID’s: 28611, 141175, 142148

**Urumaco**

Date of download: 19.04.2014

PBDB collection ID’s: 92751, 144849, 145271, 145364, 145365, 145380, 146290, 146407, 146416, 146421, 146422, 146423, 146425, 146426, 146427, 146428, 146429, 146430, 146431, 146432, 146433, 146446, 146447, 146448, 146449, 146450, 146451, 152543, 146452

**Acre**

Date of download: 28.05.2014

PBDB collection ID’s: 134808, 136714, 136716, 136717, 137876, 148207, 156532, 137877, 137878, 137879, 137880 , 137881 , 137882 , 137883, 137884 , 144064, 144066, 144515, 134868, 55602, 67383, 67384, 67385, 67386

**Mesopotamian**

Date of download: 13.04.2014

PBDB collection ID’s: 55600, 140472, 140696

**Cerro Azul**

Date of download: 04.07.2014

PBDB collection ID’s: 87198, 140925, 140932, 141939, 151498, 151499, 151500, 152063, 152065, 152066, 152067

**Chiquimil**

Date of download: 04.07.2014

PBDB collection ID: 157794

**Andalhuala**

Date of download: 04.07.2014

PBDB collection ID: 157795

**Monte Hermoso**

Date of download: 11.06.2014

PBDB collection ID’s: 13503, 140627, 141247, 145757, 152062, 152064, 152081, 152084, 152085, 152086, 152087, 152088

**Inchasi**

Date of download: 12.06.2014

PBDB collection ID: 71112

**Uquía**

Date of download: 11.06.2014

PBDB collection ID’s: Uquía lower 141894; Uquía middle 141896

1. **Supplementary Material 3**

Search queries used to get records of GABI participants in North and South America from the Paleobiology Database (PBDB).

Date of download: 16.08.2014

*NORTH AMERICAN-ORIGIN MAMMALS*

**Taxon or taxa to include**: Peradectinae, Herpetotheriinae, Peradectes, Armintodelphys , Leptictida, Lagomorpha, Alagomyidae, Laredomyidae, Ischyromyidae, Allomyidae, Aplodontoidea, Sciuroidea, Castoroidea, Castorimorpha, Cricetidae, Myodonta, Protoptychidae, Geomyoidea, Muridae, Palaeoryctidae, Cimolestidae, Apatemyidae, Taeniodonta, Tillodontia, Pantodonta, Pantolestidae, Epoicotheriidae, Metacheiromyidae, Feliformia, Amphicyonidae, Cyonidea, Lycophocyon, Palaearctonyx,Procynodictis, Canidae, Mustelida, Musteloidea,Nothocyon, Ursida, Ursidae, Creodonta, Perissodactyla, Achaenodontidae, Achtiaria,Ancodonta, Antiacodontidae, [Antiacodontinae](http://paleodb.org/?a=taxonInfo&taxon_no=67477), [Caenotheriidae](http://paleodb.org/?a=taxonInfo&taxon_no=63100), [Cetancodontamorpha](http://paleodb.org/?a=taxonInfo&taxon_no=159632), [Choeropotamoidea](http://paleodb.org/?a=taxonInfo&taxon_no=103720), [Delahomeryx](http://paleodb.org/?a=taxonInfo&taxon_no=42312), [Dichobunoidea](http://paleodb.org/?a=taxonInfo&taxon_no=57543), [Dulcidon](http://paleodb.org/?a=taxonInfo&taxon_no=42219), [Entelodontoidea](http://paleodb.org/?a=taxonInfo&taxon_no=42356), [Eolantianus](http://paleodb.org/?a=taxonInfo&taxon_no=260849), [Eschatiidae](http://paleodb.org/?a=taxonInfo&taxon_no=106437), [Eurytheriidae](http://paleodb.org/?a=taxonInfo&taxon_no=106430), [Helohyinae](http://paleodb.org/?a=taxonInfo&taxon_no=67478), [Hexacodus](http://paleodb.org/?a=taxonInfo&taxon_no=42314), [Hidrosotherium](http://paleodb.org/?a=taxonInfo&taxon_no=42315), [Hsanotherium](http://paleodb.org/?a=taxonInfo&taxon_no=58157), [Leptochoerinae](http://paleodb.org/?a=taxonInfo&taxon_no=65511), [Lophiomerycidae](http://paleodb.org/?a=taxonInfo&taxon_no=116562), [Myanmarius](http://paleodb.org/?a=taxonInfo&taxon_no=280182), [Neoselenodontia](http://paleodb.org/?a=taxonInfo&taxon_no=96404), [Nonruminantia](http://paleodb.org/?a=taxonInfo&taxon_no=147578), [Palaeodonta](http://paleodb.org/?a=taxonInfo&taxon_no=57542), [Protolabididae](http://paleodb.org/?a=taxonInfo&taxon_no=106436), [Raoellidae](http://paleodb.org/?a=taxonInfo&taxon_no=63190), [Raphenacodus](http://paleodb.org/?a=taxonInfo&taxon_no=42896), [Ruminantiamorpha](http://paleodb.org/?a=taxonInfo&taxon_no=159629), [Simpsonodus](http://paleodb.org/?a=taxonInfo&taxon_no=42322), [Suiformes](http://paleodb.org/?a=taxonInfo&taxon_no=67953), [Suoidea](http://paleodb.org/?a=taxonInfo&taxon_no=42380), [Tragulohyus](http://paleodb.org/?a=taxonInfo&taxon_no=42897), [Tylopoda](http://paleodb.org/?a=taxonInfo&taxon_no=108734), [Whippomorpha](http://paleodb.org/?a=taxonInfo&taxon_no=71831), Proboscidea, Erinaceomorpha, Soricomorpha, Solenodontidae, Talpidae, Lipotyphla, Dinocerata, Acreodi, Emballonuridae, Tadarida, Molossops, Molossus, Nyctinomops, Mormoopidae, Pteropodidae, Vespertilionidae

**Taxon or taxa to exclude:** Hystricognathi, Cavina, Pinnipedia, Cetacea, Sirenia, Desmostylia, Wangliidae, Diclidurus

**Time span:** Miocene-Holocene

**Continent:** South America

*SOUTH AMERICAN-ORIGIN MAMMALS*

**Taxon or taxa to include:** Gondwanatheria, Ameridelphia, Microbiotheria, Paucituberculata, Pucadelphydae, Didelphinae, Caluromyinae, Marmosinae, Derorhynchinae, Eobrasiliinae, Sparassocynus, Hyladelphinae, Thylamyinae, Caroloameghiniinae, Polydolopimorphia, Sparassodonta, Borhyaenoidea, Xenarthra, Hystricognathi, Cavina, Platyrrhini, Litopterna, Notoungulata, Astrapotheria, Xenungulata, Pyrotheria, Meridiungulata, Diclidurus, Furipteridae, Eumops, Kiotomops, Mormopterus, Promops, Noctilionidae, Phyllostomidae, Thyropteridae

**Taxon or taxa to exclude:** Alphadontinae, Peradectinae, Herpetotheriinae, Peradectes, Armintodelphys, Iugomortiferum, Cimolestes,

**Time span:** Miocene-Holocene

**Continent:** North America

1. **Supplementary Material 4**

The R code used to perform the different analyses

#NMDS analysis with bray-Curtis distance and average cluster dendogram

#load presence/abscence matrix from the Supplementary Table 1

Supp1<-read.csv("Supplementary_Table1.csv",row.names=1)

Supp.1<-as.matrix(Supp1)

str(Supp.1)

# load Vegan package#

library(vegan)

# Get a distance matrix for the faunas with Bray-Curtis distance

dist.Bray<-vegdist(Supp.1, method="bray", binary=TRUE)

str(dist.Bray)

# Get a cluster plot only

dist.Bray.av<-hclust(dist.Bray, method="average")

plot(dist.Bray.av,ylab="Bray curtis dissimilarity")

## Perform NMDS analysis, k value=2 and 1000 runs

nmds.data<-metaMDS(Supp.1, distance="bray",k=2, trymax=1000,autotransform=TRUE,

wascores=TRUE, expand=TRUE)

nmds.data$converge

nmds.data$stress

# Plot of the NMDS with the clusterdendrogram - Figure 4 A

dist.Bray.av.groups[]<-cutree(dist.Bray.av,k=3)

ave.lev<-levels(factor(dist.Bray.av.groups))

q<-ordiplot(nmds.data, type="n")

for(i in 1:length(ave.lev)){

points(nmds.data, col=dist.Bray.av.groups[],

pch=16,cex=2)

}

text(nmds.data,pos=3)

ordicluster(q,dist.Bray.av, col="darkgrey")

#Analysis of Bray-Curtis dissimilarity values taking into account differences in sample size

#Data for histograms showed in Figure 4C

#load taxonomic lists from Supplementary Material 1

# Middle Miocene faunal assemblages

Fitzcarrald.gen<-read.csv("Fitzcarrald.csv",sep=";")

str(Fitzcarrald.gen)

Fitzcarrald.gen$Fauna<-rep("Fitzcarrald",17)

LaVenta.gen<-read.csv("LaVenta.csv")

str(LaVenta.gen)

LaVenta.gen$Fauna<-rep("La Venta", 42)

QuebradaHonda.gen<-read.csv("QuebradaHonda.csv")

str(QuebradaHonda.gen)

QuebradaHonda.gen$Fauna<-rep("Quebrada Honda", 14)

CollonCura.gen<-read.csv("CollonCura.csv",sep=";")

str(CollonCura.gen)

CollonCura.gen$Fauna<-rep("Collon Cura", 54)

#Loops to estimate Bray-Curtis dissimilarity 1000 times for each pair of faunas

# From the fauna with more taxa (e.g. La Venta) each time is randomly chosen the same number of taxa as the #fauna with fewer taxa (e.g. Fitzcarrald)

# La Venta- Fitzcarrald - tropical middle Miocene

nrand<-1000

xy.boot =numeric(nrand)

for(i in 1:nrand){

gen<-as.vector(sample(LaVenta.gen$genus,17, replace=TRUE))

name<-rep("La Venta",17)

t<-as.data.frame(cbind(gen,name))

gen<-as.vector(Fitzcarrald.gen$genus)

name<-rep("Fitzcarrald",17)

p<-as.data.frame(cbind(gen,name))

q<-rbind(t,p)

tab<-table(q$name,q$gen)

xy.boot[i]=vegdist(tab, method="bray", binary=TRUE)

}

mean(xy.boot)

sd(xy.boot)

Lv.Fitz<-xy.boot

mean(Lv.Fitz)

#Collon Cura - Quebrada Honda - temperate middle Miocene

nrand<-1000

xy.boot =numeric(nrand)

for(i in 1:nrand){

gen<-as.vector(sample(CollonCura.gen$genus,14, replace=TRUE))

name<-rep("Collon Cura",14)

t<-as.data.frame(cbind(gen,name))

gen<-as.vector(QuebradaHonda.gen$genus)

name<-rep("Quebrada Honda",14)

p<-as.data.frame(cbind(gen,name))

q<-rbind(t,p)

tab<-table(q$name,q$gen)

xy.boot[i]=vegdist(tab, method="bray", binary=TRUE)

}

mean(xy.boot)

sd(xy.boot)

Col.Qh<-xy.boot

mean(Col.Qh)

#middle miocene temperate vs tropical

nrand<-1000

xy.boot =numeric(nrand)

for(i in 1:nrand){

gen<-as.vector(sample(CollonCura.gen$genus,14, replace=TRUE))

name<-rep("Collon Cura",14)

t<-as.data.frame(cbind(gen,name))

gen<-as.vector(sample(QuebradaHonda.gen$genus,14,replace=TRUE))

name<-rep("Quebrada Honda",14)

p<-as.data.frame(cbind(gen,name))

gen<-as.vector(sample(LaVenta.gen$genus,14, replace=TRUE))

name<-rep("La Venta",14)

o<-as.data.frame(cbind(gen,name))

gen<-as.vector(Fitzcarrald.gen$genus)

name<-rep("Fitzcarrald",14)

r<-as.data.frame(cbind(gen,name))

q<-rbind(t,p,o,r)

tab<-table(q$name,q$gen)

f<-as.matrix(vegdist(tab, method="bray", binary=TRUE))

xy.boot[i]=mean(f[1:2,3:4])

}

mean(xy.boot)

sd(xy.boot)

trop.temp.midmio<-xy.boot

mean(trop.temp.midmio)

#Acre-Urumaco, tropical late miocene

Acre.gen<-read.csv("Acre-ranges.csv")

str(Acre.gen)

Acre.gen$Fauna<-rep("Acre",53)

Urumaco.gen<-read.csv("Urumaco-ranges.csv",sep=";")

str(Urumaco.gen)

Urumaco.gen$Fauna<-rep("Urumaco", 14)

nrand<-1000

xy.boot =numeric(nrand)

for(i in 1:nrand){

gen<-as.vector(sample(Acre.gen$genus,14, replace=TRUE))

name<-rep("Acre",14)

t<-as.data.frame(cbind(gen,name))

gen<-as.vector(Urumaco.gen$genus)

name<-rep("Urumaco",14)

p<-as.data.frame(cbind(gen,name))

q<-rbind(t,p)

tab<-table(q$name,q$gen)

xy.boot[i]=vegdist(tab, method="bray", binary=TRUE)

}

mean(xy.boot)

sd(xy.boot)

Acr.Uru<-xy.boot

mean(Acr.Uru)

#temperate late miocene -Mesopotamian, Chiquimil,Andalhuala,Cerro Azul,Monte Hermoso

Mesopotamia.gen<-read.csv("Mesopotamian-ranges.csv")

str(Mesopotamia.gen)

Mesopotamia.gen$Fauna<-rep("Mesopotamian", 91)

Chiquimil.gen<-read.csv("Chiquimil-ranges.csv")

str(Chiquimil.gen)

Chiquimil.gen$Fauna<-rep("Chiquimil", 26)

Andalhuala.gen<-read.csv("Andalhuala-ranges.csv")

str(Andalhuala.gen)

Andalhuala.gen$Fauna<-rep("Andalhuala", 36)

CerroAzul.gen<-read.csv("CerroAzul-ranges.csv")

str(CerroAzul.gen)

CerroAzul.gen$Fauna<-rep("Cerro Azul", 44)

MonteHermoso.gen<-read.csv("MonteHermoso-ranges.csv")

str(MonteHermoso.gen)

MonteHermoso.gen$Fauna<-rep("Monte Hermoso", 64)

nrand<-1000

xy.boot =numeric(nrand)

for(i in 1:nrand){

gen<-as.vector(sample(Mesopotamia.gen$genus,26, replace=TRUE))

name<-rep("Mesopotamian",26)

t<-as.data.frame(cbind(gen,name))

gen<-as.vector(sample(Andalhuala.gen$genus,26,replace=TRUE))

name<-rep("Andalhuala",26)

p<-as.data.frame(cbind(gen,name))

gen<-as.vector(sample(CerroAzul.gen$genus,26, replace=TRUE))

name<-rep("CerroAzul",26)

o<-as.data.frame(cbind(gen,name))

gen<-as.vector(sample(MonteHermoso.gen$genus,26, replace=TRUE))

name<-rep("MonteHermoso",26)

s<-as.data.frame(cbind(gen,name))

gen<-as.vector(Chiquimil.gen$genus)

name<-rep("Chiquimil",26)

r<-as.data.frame(cbind(gen,name))

q<-rbind(t,p,o,s,r)

tab<-table(q$name,q$gen)

xy.boot[i]=vegdist(tab, method="bray", binary=TRUE)

}

mean(xy.boot)

sd(xy.boot)

te.lat.mio<-xy.boot

mean(te.lat.mio)

#All late miocene trop vs temp

nrand<-1000

xy.boot =numeric(nrand)

for(i in 1:nrand){

gen<-as.vector(sample(Mesopotamia.gen$genus,14, replace=TRUE))

name<-rep("Mesopotamian",14)

t<-as.data.frame(cbind(gen,name))

gen<-as.vector(sample(Andalhuala.gen$genus,14,replace=TRUE))

name<-rep("Andalhuala",14)

p<-as.data.frame(cbind(gen,name))

gen<-as.vector(sample(CerroAzul.gen$genus,14, replace=TRUE))

name<-rep("CerroAzul",14)

o<-as.data.frame(cbind(gen,name))

gen<-as.vector(sample(MonteHermoso.gen$genus,14, replace=TRUE))

name<-rep("MonteHermoso",14)

s<-as.data.frame(cbind(gen,name))

gen<-as.vector(sample(Chiquimil.gen$genus,14,replace=TRUE))

name<-rep("Chiquimil",14)

r<-as.data.frame(cbind(gen,name))

gen<-as.vector(sample(Acre.gen$genus,14, replace=TRUE))

name<-rep("Acre",14)

w<-as.data.frame(cbind(gen,name))

gen<-as.vector(Urumaco.gen$genus)

name<-rep("Urumaco",14)

z<-as.data.frame(cbind(gen,name))

q<-rbind(t,p,o,s,r,w,z)

tab<-table(q$name,q$gen)

f<-as.matrix(vegdist(tab, method="bray", binary=TRUE))

xy.boot[i]= mean(f[1:5,6:7])

}

mean(xy.boot)

sd(xy.boot)

lat.mio<-xy.boot

mean(lat.mio)

#Mann U test for the dissimilarity values of the tropical and temperate middle and late Miocene faunas

wilcox.test(Lv.Fitz,trop.temp.midmio)

wilcox.test(Col.Qh, trop.temp.midmio)

wilcox.test(Acr.Uru,lat.mio)

wilcox.test(te.lat.mio,lat.mio)

# Analysis of cumulative first appearance (FAD) during the GABI in tropics vs temperate – Data for figure 6A

# Load First appearance data from Supplementary Table 2

all.fad<-read.csv(Supplementary_Table2)

#Separate tropical and temperate fad

trop.fad<-subset(all.fad, paleolatdec<=23 & paleolatdec>= -23)

str(trop.fad)

plot(trop.fad$ma_mid, trop.fad$paleolatdec)

temp.fad<-subset(all.fad, paleolatdec< -23 | paleolatdec> 23)

str(temp.fad)

plot(temp.fad$ma_mid, temp.fad$paleolatdec)

# Generate the resampling of the age of each fad record given their estimated age range (max and min age)

# The function for the loop

fun1<-function(df,i,N){runif(N,min=df$ma_min[i], max=df$ma_max[i])}

trop.resam<-foreach(i= 1:67)%do% fun1(trop.fad,i,1000)

# For the tropics

trop.fad$mean.resample<-as.numeric(foreach(i= 1:67)%do%

mean(fun1(trop.fad,i,1000)))

trop.fad$sd.resample<-as.numeric(foreach(i= 1:67)%do%

sd(fun1(trop.fad,i,1000)))

#Calculate the mean and standard deviation (sd) for the fad in the tropics

str(trop.fad)

tropics.mean<-trop.fad$mean.resample

tropics.sd1<-(trop.fad$mean.resample+trop.fad$sd.resample)

tropics.sd2<-(trop.fad$mean.resample - trop.fad$sd.resample)

tropics.meancut<-cut(tropics.mean,breaks, right=TRUE)

tropics.meanfreq<-table(tropics.meancut)

tropics.sd1cut<-cut(tropics.sd1,breaks, right=TRUE)

tropics.sd1freq<-table(tropics.sd1cut)

tropics.sd2cut<-cut(tropics.sd2,breaks, right=TRUE)

tropics.sd2freq<-table(tropics.sd2cut)

trop.meancumfreq<-c(0,cumsum(rev(tropics.meanfreq)))

trop.sd1cumfreq<-c(0,cumsum(rev(tropics.sd1freq)))

trop.sd2cumfreq<-c(0,cumsum(rev(tropics.sd2freq)))

#For temperate fad

temp.fad$mean.resample<-as.numeric(foreach(i= 1:52)%do%

mean(fun1(temp.fad,i,1000)))

temp.fad$sd.resample<-as.numeric(foreach(i= 1:52)%do%

sd(fun1(temp.fad,i,1000)))

#Calculate mean and standard deviation sd for the fad in temperate

str(temp.fad)

temp.mean<-temp.fad$mean.resample

temp.sd1<-(temp.fad$mean.resample+temp.fad$sd.resample)

temp.sd2<-(temp.fad$mean.resample - temp.fad$sd.resample)

breaks2<-seq(15,0,by= -1)

temp.meancut<-cut(temp.mean,breaks2, right=TRUE)

temp.meanfreq<-table(temp.meancut)

temp.sd1cut<-cut(temp.sd1,breaks2, right=TRUE)

temp.sd1freq<-table(temp.sd1cut)

temp.sd2cut<-cut(temp.sd2,breaks2, right=TRUE)

temp.sd2freq<-table(temp.sd2cut)

temp.meancumfreq<-c(0,cumsum(rev(temp.meanfreq)))

temp.sd1cumfreq<-c(0,cumsum(rev(temp.sd1freq)))

temp.sd2cumfreq<-c(0,cumsum(rev(temp.sd2freq)))

# Plot for the fad during GABI shown in Figure 6A

plot(breaks,trop.meancumfreq, xlim=c(12,0), type="b", col="red", pch=16,

xlab="Time (ma)", ylab= "Cumulative FAD", ylim=c(0,100))

segments(breaks,trop.meancumfreq,breaks,trop.sd1cumfreq,col="red",lty=2)

segments(breaks,trop.meancumfreq,breaks,trop.sd2cumfreq,col="red",lty=2)

points(breaks,temp.meancumfreq, type="b", col="blue", pch=16)

segments(breaks,temp.meancumfreq,breaks,temp.sd1cumfreq,col="blue",lty=2)

segments(breaks,temp.meancumfreq,breaks,temp.sd2cumfreq,col="blue",lty=2)
